# Characterization of Informed Consent Forms Posted on ClinicalTrials.gov

**DOI:** 10.1001/jamanetworkopen.2021.35146

**Published:** 2021-11-18

**Authors:** Tony Tse, Sarah White, Luke Gelinas, Walker Morrell, Barbara Bierer, Deborah A. Zarin

**Affiliations:** 1 National Center for Biotechnology Information, National Library of Medicine, National Institutes of Health, Bethesda, Maryland; 2 Multi-Regional Clinical Trials Center of Brigham and Women’s Hospital and Harvard, Cambridge, Massachusetts; 3 Advarra, Columbia, Maryland

## Abstract

This cross-sectional study examines available forms and posting trends of registered trials as well as the frequency of form posting by funder type for trials initiated since the revised Common Rule was implemented.

## Introduction

Informed consent forms (hereinafter, *forms*), part of a larger consent process that serves multiple bioethical functions,^1^ are intended to provide potential research volunteers with sufficient written information about a clinical trial to help them decide about participation. Despite concerns about their overall quality, broadly generalizable samples of forms have been difficult to access for quality improvement.^2,3^ Since July 2017, ClinicalTrials.gov has allowed voluntary posting of forms^4^ for registered studies. Subsequently (January 21, 2019), the revised Common Rule form-posting requirement (45 CFR 46.116[h])^5^ became effective (eAppendix in the Supplement). To explore how access to forms has increased on ClinicalTrials.gov after these initiatives, we sought to characterize registered trials with available forms and posting trends. We also assessed the frequency of form posting by funder type for trials initiated since the revised Common Rule compliance date.

## Methods

We conducted cross-sectional analyses using 2 data sets downloaded from ClinicalTrials.gov on July 7, 2021. Set 1 was used to characterize all registered clinical trials with at least 1 US site and a posted form. Set 2 consisted of registered US trials with start dates on or after January 21, 2019; study completion dates on or before June 30, 2021; and with or without a posted form. We assessed the percentage of trials with a posted form by funder type among registered trials initiated since the revised Common Rule general compliance date. Institutional review board approval was not required because this study did not involve any human participants (and only assessed publicly posted clinical trial records). This report followed the Strengthening the Reporting of Observational Studies in Epidemiology (STROBE) reporting guideline.

## Results

Of 2088 trials with forms posted on ClinicalTrials.gov (set 1), 986 (47.2%) listed funding only by “other” nonfederal and nonindustry sources (eg, foundations) while another 846 (40.5%) listed funding by US federal agencies (including the National Institutes of Health [NIH]) (Table). Furthermore, 976 trials (46.7%) listed at least 1 drug, biologic, or genetic intervention; 468 (22.4%) listed at least 1 device, diagnostic, or radiation intervention (ie, likely products regulated by the US Food and Drug Administration); 1520 (72.8%) had completed or terminated enrollment; 1050 (50.3%) had 0 to 50 participants; and the most listed conditions were obesity, depression, prostate cancer, breast cancer, and stroke. In addition, 1972 (94.4%) of set 1 trials were associated with 542 nonindustry sponsors, and the remaining 116 (5.6%) were associated with 85 industry sponsors. The Figure shows the cumulative number of forms available at ClinicalTrials.gov for set 1 trials.

**Table.** Selected Characteristics of Data Set 1: 2088 Registered US Clinical Trials With Posted Informed Consent Forms on ClinicalTrials.gov

Characteristic	Records, No (%)
**Trial attribute**	
Key funder type^a^	
NIH	697 (33.4)
Other federal agency	148 (7.1)
Industry	257 (12.3)
Other (eg, foundation)	986 (47.2)
Current overall recruitment status	
Active, not recruiting	204 (9.8)
Completed	1271 (60.9)
Enrolling by invitation	26 (1.2)
Not yet recruiting	18 (0.9)
Recruiting	279 (13.4)
Suspended	9 (0.4)
Terminated	249 (11.9)
Unknown status	27 (1.3)
Withdrawn	5 (0.2)
Enrollment (anticipated or actual), No. of participants	
0-50	1050 (50.3)
51-100	399 (19.1)
101-500	509 (24.4)
>500	130 (6.2)
Intervention type, at least 1 of following	
Behavioral	537 (25.7)
Device, diagnostic, or radiation	468 (22.4)
Medical procedure	130 (6.2)
Dietary supplement, combination, other	457 (21.9)
Drug, biologic, or genetic	976 (46.7)
Not applicable	56 (5.7)
Early phase 1 or phase 1	138 (14.1)
Phase 1/2 or phase 2	481 (49.3)
Phase 2/3 or phase 3	124 (12.7)
Phase 4	177 (18.1)
Top 5 conditions, at least 1 of the following	
Obesity	47 (2.3)
Depression	45 (2.2)
Prostate cancer	39 (1.9)
Breast cancer	33 (1.6)
Stroke	31 (1.5)
Study design	
Not provided	3 (0.1)
1 Arm	563 (27.0)
>1 Arm	1522 (72.9)
Randomized allocation	1324 (87.0)
**Lead sponsor**	
Industry (85 sponsors)	116 (5.6)
No. of trials per sponsor	
Mean (range)	1.4 (1-7)
Median (IQR)	1 (1-1)
Nonindustry (542 sponsors)	1972 (94.4)
No. of trials per sponsor	
Mean (range)	3.6 (1-132)
Median (IQR)	1 (1-3)

Abbreviation: NIH, National Institutes of Health.

^a^ Key funder types were categorized as “NIH” if at least 1 NIH institute or center was listed as the lead sponsor or collaborator, as “other federal agency” if not classified as NIH and at least 1 federal agency (other than NIH) or department was listed as the lead sponsor or collaborator, as “industry” if not classified as either NIH or other federal agency and at least 1 company was listed as the lead sponsor or collaborator, and as “other” for all remaining records (eg, foundation funding).

**Figure.**
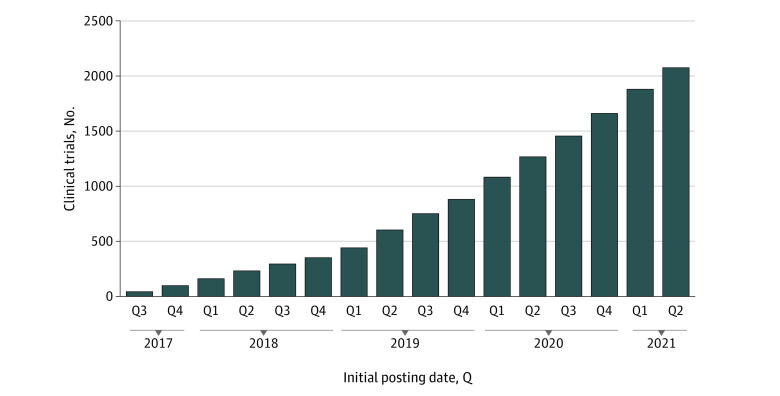
Cumulative US Clinical Trials With Informed Consent Forms Posted on ClinicalTrials.gov, by Initial Posting Date Numbers of registered trials by quarter (Q) from 2017 Q3 (n = 42) to 2021 Q2 (n = 2076). The 12 trials for which informed consent forms were posted from July 1 through 7, 2021 (ie, 2021 Q3), were omitted from this figure. The option to submit informed consent documents to ClinicalTrials.gov became available on June 29, 2017. Sponsors and investigators may submit informed consent forms to ClinicalTrials.gov for posting at any time during the study life cycle.

Among 4754 registered trials in set 2 (ie, initiated since the revised Common Rule compliance date), trials with posted forms by key funder type were 17.7% (71 of 401) funded by the NIH, 12.5% (16 of 128) funded by another US federal agency, 5.1% (113 of 2209) funded by another organization (eg, foundation), and 0.9% (19 of 2016) funded by industry. Overall, the percentage of set 2 trials with posted forms was 4.6% (219 of 4574).

## Discussion

As of July 7, 2021, forms were publicly available on ClinicalTrials.gov for nearly 2100 US trials for a range of intervention types and conditions from across 600 mostly nonindustry sponsors. Many of these trials (1243 of 2088 [59.5%]) did not list funding by a US federal agency and, among those 1243 trials, some were initiated before the compliance date, suggesting that their forms were likely not required to be posted under the revised Common Rule.

The absolute percentages of federally funded trials initiated since the Common Rule compliance date in set 2 remain relatively low, with fewer than 87 of 529 trials (16.5%) listing a key funder type of “NIH” or “other US federal agency” having posted forms. Although forms for a range of trials are now available on ClinicalTrials.gov, most appear to have been posted voluntarily.

Limitations of this cross-sectional study include that retrieved trials were likely skewed toward those required by federal reporting requirements. Trials may have also been miscategorized because of errors or incomplete information in data self-reported by study sponsors. Further research is needed because it is likely too soon to assess the full impact of the revised Common Rule requirement.

## Notes

Supplement. **eAppendix.** Revised Common Rules for Clinical Trials
eReferences
