# Supplementary material for: Characterization of Informed Consent Forms Posted on ClinicalTrials.gov
Source: JAMA Netw Open. 2021 Nov 18;4(11):e2135146. doi: 10.1001/jamanetworkopen.2021.35146 (PMC8603088; doi:10.1001/jamanetworkopen.2021.35146)
Supplement: Supplement. — eAppendix. Revised Common Rules for Clinical Trials eReferences [file jamanetwopen-e2135146-s001.pdf]

Supplemental Online Content

Tse T, White S, Gelinas L, Morrell W, Bierer B, Zarin DA. Characterization of informed consent forms posted on ClinicalTrials.gov. *JAMA Netw Open*. 2021;4(11):e2135146. doi:10.1001/jamanetworkopen.2021.35146

eAppendix. Revised Common Rules for Clinical Trials

eReferences

This supplemental material has been provided by the authors to give readers additional information about their work.

© 2021 Tse T et al. *JAMA Network Open.*

eAppendix. Revised Common Rules for Clinical Trials

A. Selected Regulatory Provisions: Revised Common Rule (45 CFR 46)[1]

45 CFR 46.116(h) ***Posting of clinical trial consent form***. (1) For each clinical trial conducted or supported by a Federal department or agency, one IRB-approved informed consent form used to enroll subjects must be posted by the awardee or the Federal department or agency component conducting the trial on a publicly available Federal Web site that will be established as a repository for such informed consent forms.

(2) If the Federal department or agency supporting or conducting the clinical trial determines that certain information should not be made publicly available on a Federal Web site (e.g. confidential commercial information), such Federal department or agency may permit or require redactions to the information posted.

(3) The informed consent form must be posted on the Federal Web site after the clinical trial is closed to recruitment, and no later than 60 days after the last study visit by any subject, as required by the protocol.

45 CFR 46.101(l)(2) ***2018 Requirements***. For purposes of this section, the 2018 Requirements means the Federal Policy for the Protection of Human Subjects requirements contained in this subpart. The general compliance date for the 2018 Requirements is January 21, 2019. The compliance date for §46.114(b) (cooperative research) of the 2018 Requirements is January 20, 2020.

B. Excerpt from "Federal Websites That Will Satisfy the Revised Common Rule's Requirement to Post Clinical Trial Consent Forms"[2]

At this time two publicly available federal websites that will satisfy the consent form posting requirement, as required by the revised Common Rule, have been identified: ClinicalTrials.gov and a docket folder on Regulations.gov (Docket ID: HHS-OPHS-2018-0021). HHS and other Common Rule departments and agencies are developing instructions and other materials providing more information to the regulated community about this posting requirement.

© 2021 Tse T et al. *JAMA Network Open.*

eReferences

1. U.S. Federal Register. Federal Policy for the Protection of Human Subjects. 2017 Jan 19. 82 FR 7149. Available at https://www.federalregister.gov/documents/2017/01/19/2017-01058/federal-policy-for-the-protection-of-human-subjects. Last accessed on August 8, 2021.

2. U.S. Department of Health and Human Services. Announcement: Federal websites that will satisfy the revised Common Rule’s requirement to post clinical trial consent forms (45 CFR 46.116(h)). Aug 15, 2018. HHS-OPHS-2018-0021-0001. Available at https://downloads.regulations.gov/HHS-OPHS-2018-0021-0001/content.pdf. Last accessed on August 8, 2021.

© 2021 Tse T et al. *JAMA Network Open.*
